# MiR-146a-5p inhibits cell proliferation and cell cycle progression in NSCLC cell lines by targeting CCND1 and CCND2

**DOI:** 10.18632/oncotarget.11040

**Published:** 2016-08-03

**Authors:** Yan-Li Li, Ju Wang, Cai-Yan Zhang, Yu-Qing Shen, Hui-Min Wang, Lei Ding, Yu-Chen Gu, Jia-Tao Lou, Xin-Tai Zhao, Zhong-Liang Ma, You-Xin Jin

**Affiliations:** ^1^ School of Life Sciences, Shanghai University, Shanghai 200444, China; ^2^ Department of Laboratory Medicine, Shanghai Chest Hospital Affiliated to Shanghai Jiaotong University, Shanghai 200030, China; ^3^ Shanghai Shines Pharmaceuticals Co., Ltd., Shanghai 200032, China

**Keywords:** miR-146a-5p, non-small cell lung cancer, tumor suppressor gene, cell cycle, CCND1

## Abstract

Previous studies have indicated that miR-146a-5p acts as an oncogene in several types of cancer, yet a tumor suppressor gene in others. In non-small cell lung cancer (NSCLC), one report showed that it was downregulated and played the role of tumor suppressor. However, another study showed that miR-146a-5p was overexpressed in the serum of NSCLC patients compared to healthy controls. Therefore, it is obvious that further study of the function of miR-146a-5p in NSCLC is necessary to fully understand its importance. Herein, we have verified that miR- 146a- 5p acts as a tumor suppressor in NSCLC. Our data revealed that the expression level of miR-146a-5p was significantly decreased in several human NSCLC cell lines, and also less abundant in human NSCLC tissues, when compared with controls. Moreover, we observed that miR-146a-5p could suppress cell proliferation, both *in vitro* and *in vivo*. Our results also showed that miR-146a-5p directly targeted the 3′-UTR of CCND1 and CCND2 mRNAs as well as decreased their expression at both mRNA and protein levels, causing cell cycle arrest at the G0/G1 phase. Furthermore, siRNA-mediated downregulation of CCND1 or CCND2 yielded the same effects on proliferation and cell cycle arrest as miR-146a-5p upregulation did in the NSCLC cell lines. We confirmed that the expression of miR-146a-5p had negative relationship with CCND1 or CCND2. Besides, we also found that miR-146a-5p could inhibit tumor growth in xengroft mouse models, and CCND1 and CCND2 were downregulated in miR-146a-5p overexpressed xengroft tumor tissues. In summary, our results demonstrated that miR-146a-5p could suppress the proliferation and cell cycle progression in NSCLC cells by inhibiting the expression of CCND1 and CCND2.

## INTRODUCTION

Lung cancer is the leading cause of cancer-related mortality worldwide in both men and women, responsible for more deaths than breast, colon, and prostate tumors [1]. Non-small cell lung cancer (NSCLC) accounts for about 80% of all lung cancers, and the 5-year survival rate is still very poor [2]. Therefore it is imperative to identify better therapeutic targets for the treatment of human NSCLC. Recently, an increasing number of studies have shown that microRNAs (miRNAs) are involved in NSCLC pathogenesis, providing important new insights into lung tumor biology [3–7].

miRNAs are a class of single stranded small non-coding RNAs, 18–25 nucleotides (nt) in length, which exist widely in the eukaryotic organisms [8]. They regulate gene expression on the post-transcriptional level via binding to the 3′-untranslated region (3′-UTR) of the target mRNA, resulting in either its degradation or translational repression [9, 10]. Recent evidence has demonstrated that aberrant expression of miRNAs is a critical factor influencing various cellular processes [11], thus identifying these miRNAs as either tumor suppressors or oncogenes in various cancer types, including NSCLC [10, 12].

Some studies have implicated miR-146a-5p as an oncogenic miRNA in papillary thyroid carcinoma (PTC) [13, 14] and anaplastic thyroid cancer (ATC) [15], yet carcinostatic miRNA in breast cancer [16], prostate cancer [17–19], pancreatic cancer [20], and gastric cancer [21–25]. Therefore, the role of miR-146a-5p can vary in different types of cancers. One study has shown that miR-146a-5p was downregulated in NSCLC cells (H358, H1650 and H1975) compared with non-tumor tissues and could inhibit cell growth and cell migration, as well as induce apoptosis by targeting the EGFR gene [26]. Another study showed that miR-146a-5p was overexpressed in the serum of NSCLC patients compared with healthy controls [27]. Thus, it can be concluded that miR-146a-5p has an important and interesting function in NSCLC, warranting further study.

It has been reported that the Kras oncogene is frequently mutated in NSCLC, resulting in the activation of the mitogen-activated protein kinase (MAPK) pathway [28]. We used Solexa sequencing to determine the miRNA expression profile in lung tissues of NSCLC models (L822T1/Kras^+/+^, L903T1/Kras^+/+^+LKB1^−/−^ and L703T2/Kras^+/+^+p53^−/−^) and normal mouse (L1805), and found that miR-146a-5p was significantly downregulated in NSCLC, compared to the normal control (Supplementary Figure S1). To further study the function of miR-146a-5p in NSCLC, bioinformatics analysis was used to predict potential targets, the results from which revealed hundreds of mRNAs matching the miR-146a-5p seed sequence, including cell cycle factors CCND1 and CCND2, which are downstream molecules regulated by MAPK pathway.

In the present study, we first detected the expression of miR-146a-5p in NSCLC cell lines and tissues. We then established miR-146a-5p stably overexpressing H1299 and SPCA-1 cell lines in order to analyze the biological functions of miR-146a-5p, as well as the post-transcriptional regulatory relationship between miR- 146a- 5p and CCND1 and CCND2 in NSCLC cells. Our results demonstrated that miR-146a-5p functioned as a tumor suppressor gene in NSCLC cells, and may therefore provide a potential molecular therapeutic target for human NSCLC.

## RESULTS

### MiR-146a-5p is downregulated in NSCLC tissues and human cell lines

**Figure 1:**
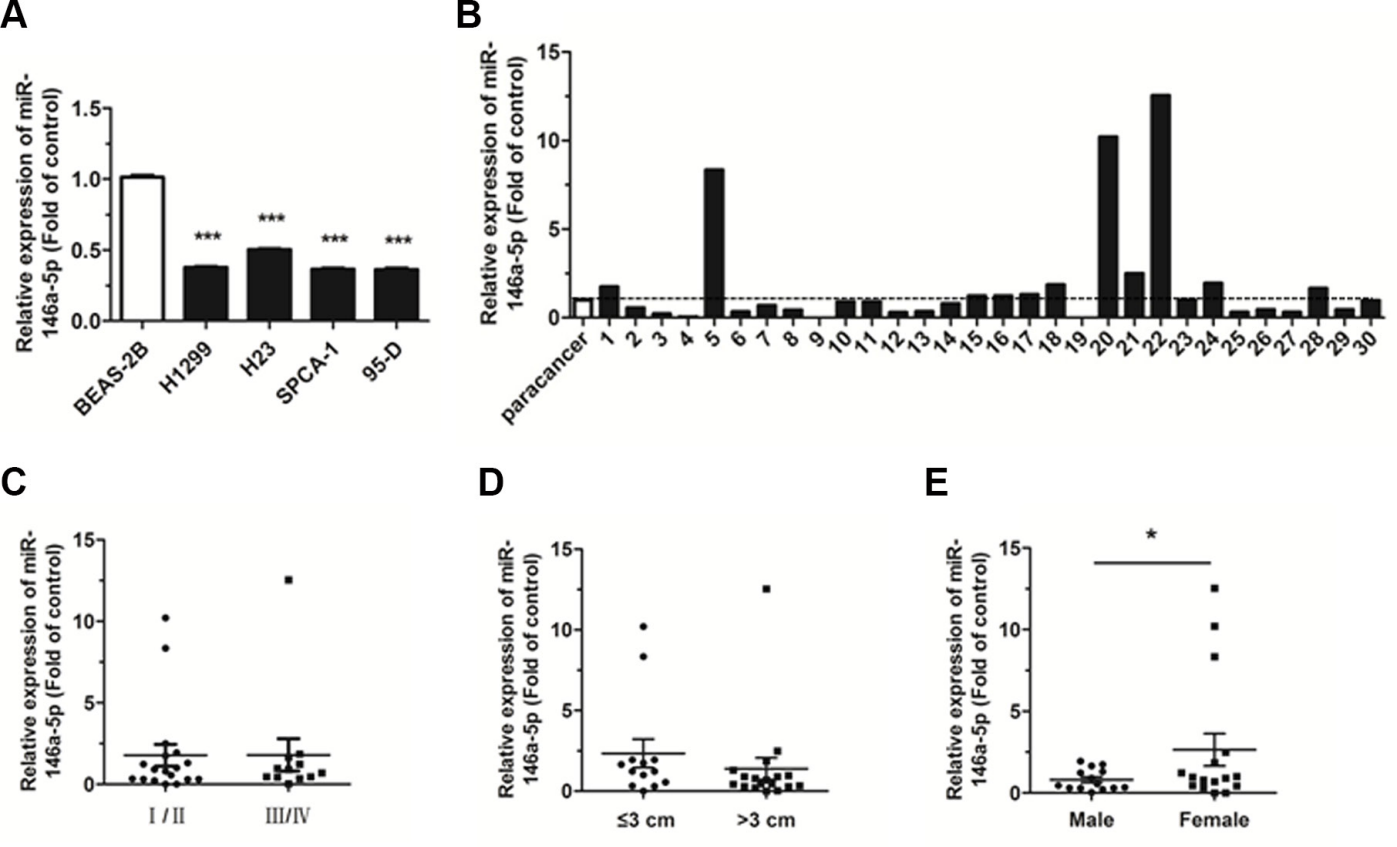
Expression of miR-146a-5p decreased in NSCLC tissues and cell lines (**A**) The expression of miR-146a-5p in the human NSCLC cell lines, H1299, H23, SPCA-1, and 95-D. The expression of miR-146a-5p in BEAS-2B cells was used for the normal control. (**B**) Relative expression of miR-146a-5p in NSCLC and corresponding paracancerous lung tissues (*n* = 30). (**C–E**) Expression of miR-146a-5p as related to pathological stage, tumor size, and sex. *P < 0.05, ***P < 0.001.

The expression level of miR-146a-5p was detected in four human NSCLC cell lines by quantitative real-time PCR (qRT-PCR). Results showed that, compared with the normal bronchial epithelium cells, BEAS-2B, miR-146a- 5p was significantly downregulated in all four NSCLC cell lines (Figure 1A). The expression level of miR- 146a- 5p was further analyzed in 30 human NSCLC samples and corresponding para-carcinoma tissues. This analysis revealed that miR-146a-5p was downregulated in 18 of the 30 cancerous tissues, when compared with corresponding paracancerous tissues (Figure 1B). Although the downregulation of miR-146a-5p was not associated with pathological stage (Figure 1C), it was closely related to both tumor size (Figure 1D) and sex (Figure 1E). The miR-146a-5p expression level in tissue samples from tumors > 3 cm in size was lower than that in tissue samples ≤ 3 cm. There was also a significant decrease of miR-146a-5p expression in the tissue samples from male patients compared with those from female patients. These results indicated that downregulation of miR-146a-5p may play an important role in the progression and development of NSCLC.

### MiR-146a-5p inhibits cell proliferation, colony formation and impedes cell cycle progression in NSCLC cell lines

**Figure 2:**
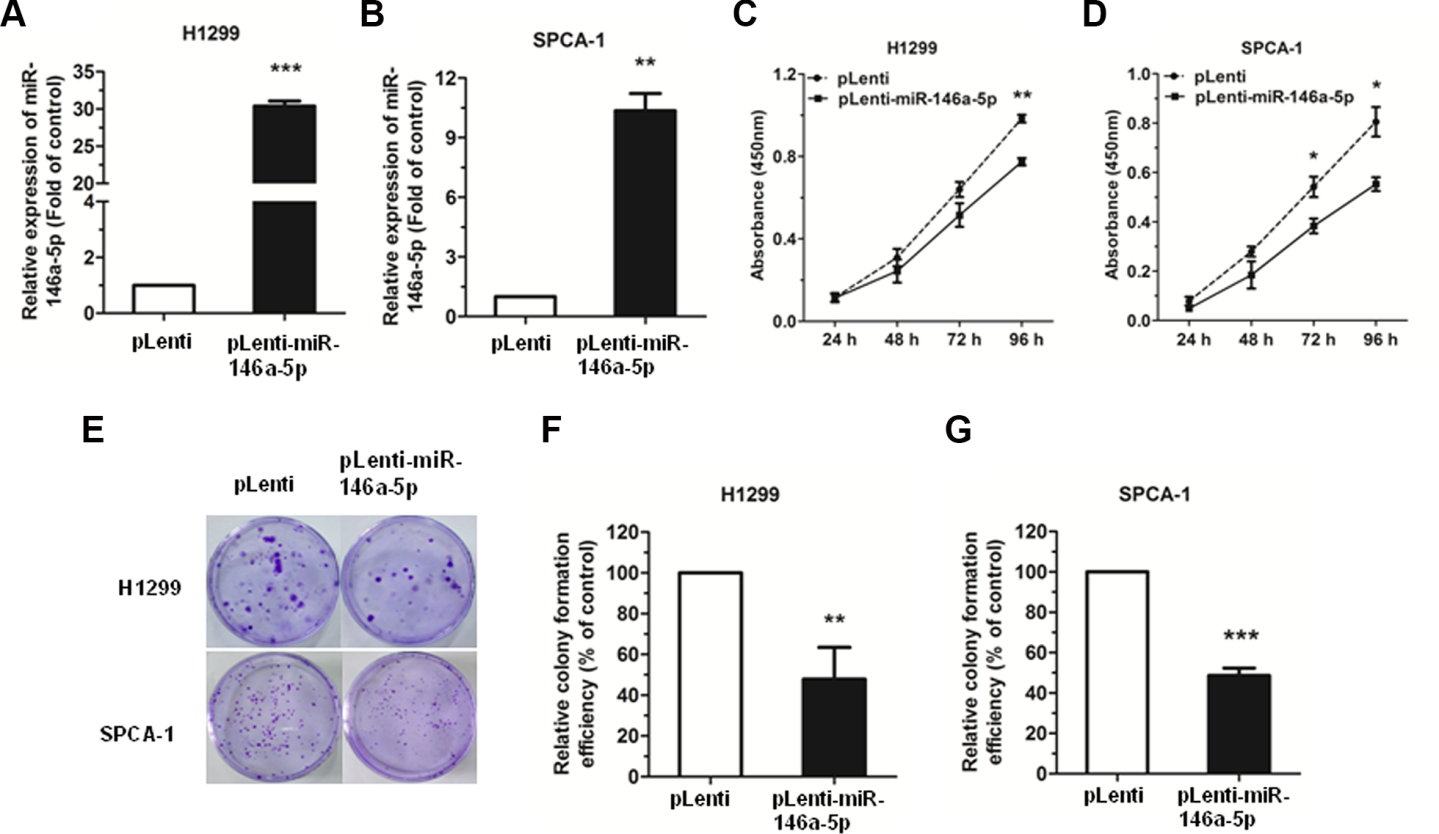
miR-146a-5p could inhibit cell proliferation and colony formation in NSCLC cell lines (**A–B**) Upregulation of miR-146a-5p in miR-146a-5p-stably-overexpressing H1299 and SPCA-1 cells. (**C–D**) The proliferation of miR-146a-5p-stably-overexpressing H1299 and SPCA-1 cells (pLenti-miR-146a-5p) and their controls (pLenti) was determined by CCK-8 assay. (**E**) Colony formation assay of miR-146a-5p-stably-overexpressing H1299 and SPCA-1 cells and their controls. (**F–G**) Relative colony formation efficiency in miR-146a-5p-stably-overexpressing H1299 and SPCA-1 cells compared to their controls. All experiments were repeated in triplicate. ^*^P < 0.05, ^**^P < 0.01, ^***^P < 0.001.

The expression level of miR-146a-5p was significantly upregulated in miR-146a-5p-stably-overexpressing (pLenti-miR-146a-5p) H1299 and SPCA-1 cell lines, as compared with negative control (NC) group (pLenti), with approximately a 200 and 10 fold increase, respectively (Figure 2A, 2B). The percentage of cells positive for green fluorescence was nearly 99% in both the control and the miR-146a-5p-stably-overexpressing H1299 and SPCA-1 cell lines (Supplementary Figure S2A, S2B).

The effect of miR-146a-5p on the proliferation of NSCLC cells *in vitro* was examined by Cell Counting Kit-8 (CCK-8) assay. Results showed that there was a significant decrease in the absorbance in the miR-146a-5p-stably-overexpressing H1299 or SPCA-1 cells when compared with the NC group (Figure 2C, 2D). Together, these data indicated that miR-146a-5p could inhibit the proliferation of NSCLC cell lines.

We further examined the effects of miR-146a- 5p on the ability of H1299 and SPCA-1 cells to form colonies, and found that miR-146a-5p could significantly inhibit the colony formation in the miR-146a-5p-stably-overexpressing H1299 or SPCA-1 cells, when compared with the NC group (Figure 2E–2G).

**Figure 3:**
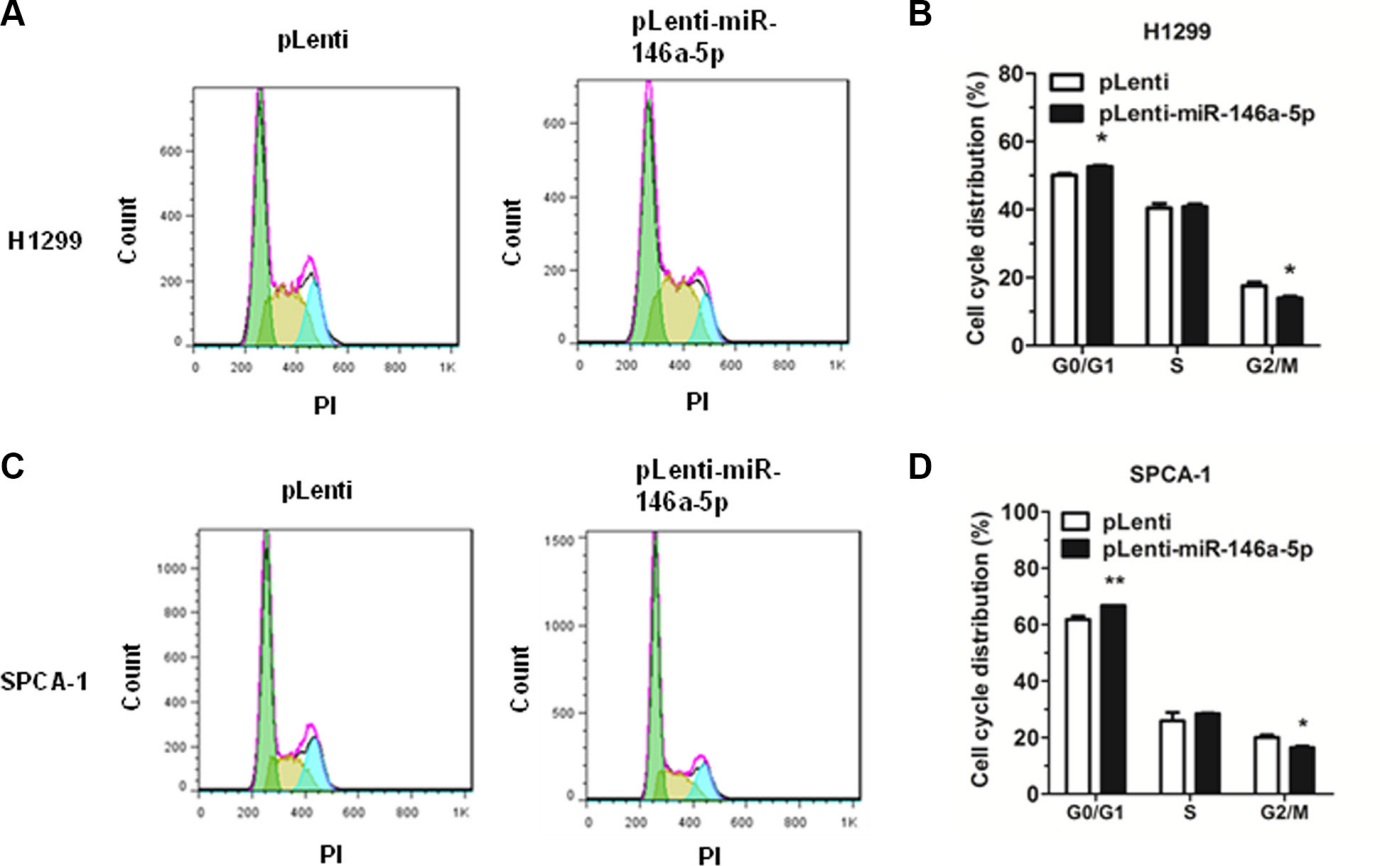
miR-146a-5p inhibited cell cycle progression in NSCLC cell lines Cell cycle analysis was performed on H1299 and SPCA-1 cells using PI to stain DNA prior to flow cytometry. (**A-B**) Cell cycle distribution of miR-146a-5p-stably-overexpressing H1299 cells and its control. (**C-D**) Cell cycle distribution of miR-146a-5p-stably-overexpressing SPCA-1 cells (pLenti-miR-146a-5p) and its control (pLenti). All experiments were repeated in triplicate. ^*^P < 0.05, ^**^P < 0.01.

Additionally, cell cycle analysis was performed in H1299 and SPCA-1 cells through the staining of DNA with propidium iodide (PI) prior to flow cytometry. Results showed that, in the NSCLC cell lines H1299 and SPCA-1, miR-146a-5p could inhibit cell cycle progression via G0/G1 arrest (Figure 3A, 3C). Cell cycle distribution was also analyzed (Figure 3B, 3D).

### MiR-146a-5p directly targets CCND1 and CCND2

**Figure 4:**
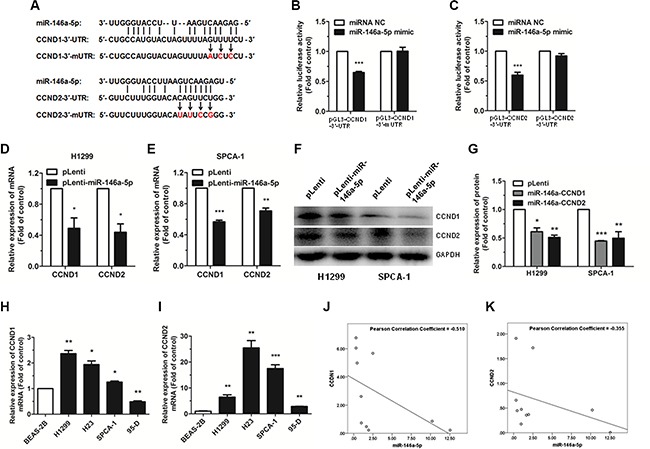
miR-146a-5p targets CCND1 and CCND2 in NSCLC cells (**A**) A schematic of the seed region of miR-146a- 5p in the 3′-UTR of CCND1 and CCND2 (CCND1-3′-UTR and CCND2-3′-UTR) and the mutated 3′-UTRs (CCND1-3′-mUTR and CCND2-3′-mUTR) as predicted by bioinformatics. (**B–C**) There was a significant decrease in the luciferase activity of HEK293T cells co-transfected with miR-146a-5p mimic and pGL3-CCND1-3′-UTR or pGL3-CCND2-3′-UTR. (**D–E**) The expression of CCND1 and CCND2 mRNA was detected by qRT-PCR in miR-146a-5p-stably-overexpressing H1299 and SPCA-1 cells (pLenti-miR-146a-5p) and their controls (pLenti). 18S RNA was used as an internal reference. (**F**) The expression of CCND1 and CCND2 protein was detected by western blotting in miR- 146a-5p-stably-overexpressing H1299 and SPCA-1 cells and their controls. Each assay was performed in triplicate. (**G**) The relative expression of proteins in miR-146a-5p-stably-overexpressing cells (miR-146a-CCND1 and miR- 146a-CCND2) and the control (pLenti) was quantified by densitometry after normalization to GAPDH. **(H–I**) The expression of CCND1 and CCND2 mRNA was detected by qRT-PCR in NSCLC cell lines. (**J–K**) MiR-146a-5p had a negative correlation with CCND1 and CCND2 according to Pearson Correlation Coefficient. ^*^P < 0.05, ^**^P < 0.01, ^***^P < 0.001.

To explore the molecular mechanism of the miR- 146a-5p-mediated G0/G1 phase cell cycle arrest in NSCLC cells, potential targets were predicted with StarBase (http://starbase.sysu.edu.cn/). CCND1 and CCND2 were chosen for further analysis, due to their important function in the regulation of cell cycle progression. The wild type binding sites and the mutation binding sites of miR-146a-5p with CCND1 and CCND2 are displayed in Figure 4A. In order to verify these targeting relationships, we constructed four recombinant expression vectors containing the miR-146a-5p wild type binding sequences in the 3′-UTR of CCND1 and CCND2 and their mutations (pGL3-CCND1-3′-UTR, pGL3-CCND2-3′-UTR, pGL3-CCND1-3′-mUTR, and pGL3-CCND2-3′-mUTR), and co-transfected them along with pRL vector and miR-146a-5p mimic or miRNA NC in HEK293T cells. The relative luciferase activity of the reporter gene was significantly decreased in the HEK293T cells co-transfected with pGL3-CCND1-3′-UTR or pGL3-CCND2-3′-UTR and miR-146a-5p mimic by 50% and 30% compared to the control (co-transfected with pGL3-CCND1-3′-UTR or pGL3-CCND2-3′-UTR and miRNA NC), whereas the relative luciferase activity of the reporter gene in the HEK293T cells co-transfected with pGL3-CCND1-3′-mUTR or pGL3-CCND2-3′-mUTR and miR-146a-5p mimic was no different with the control (co-transfected with pGL3-CCND1-3′-mUTR or pGL3-CCND2-3′-mUTR and miRNA NC) (Figure 4B, 4C). Our results demonstrated that there was a miR-146a-5p binding site in the 3′-UTR of CCND1 or CCND2.

To further investigate if miR-146a-5p decreased CCND1 and CCND2 expression at both the transcriptional and translational levels in NSCLC cells, we performed qRT-PCR and western blotting to determine the mRNA and protein levels of CCND1 and CCND2. Our results showed that the mRNA and protein expression levels of both CCND1 and CCND2 were significantly decreased in the miR-146a-5p-stably-overexpressing H1299 and SPCA-1 cells, compared with the control (Figure 4D–4G).

The expression level of CCND1 and CCND2 mRNA in human NSCLC cell lines and tissue samples was also evaluated. Results showed that CCND1 and CCND2 were significantly upregulated in the NSCLC cell lines compared with the non-pathogenic BEAS- 2B cells (except CCND1 in 95-D) (Figure 4H, 4I). According to the analysis of Pearson Correlation Coefficient, miR-146a- 5p had a negative correlation with CCND1 and CCND2 in human NSCLC tissue samples. (Figure 4J, 4K).

Taken together, these results indicated that miR- 146a-5p could directly target CCND1 and CCND2 by interacting with the 3′-UTR in NSCLC cell lines.

### Downregulation of CCND1 and CCND2 inhibits cell proliferation and cell cycle progression in NSCLC cell lines

**Figure 5:**
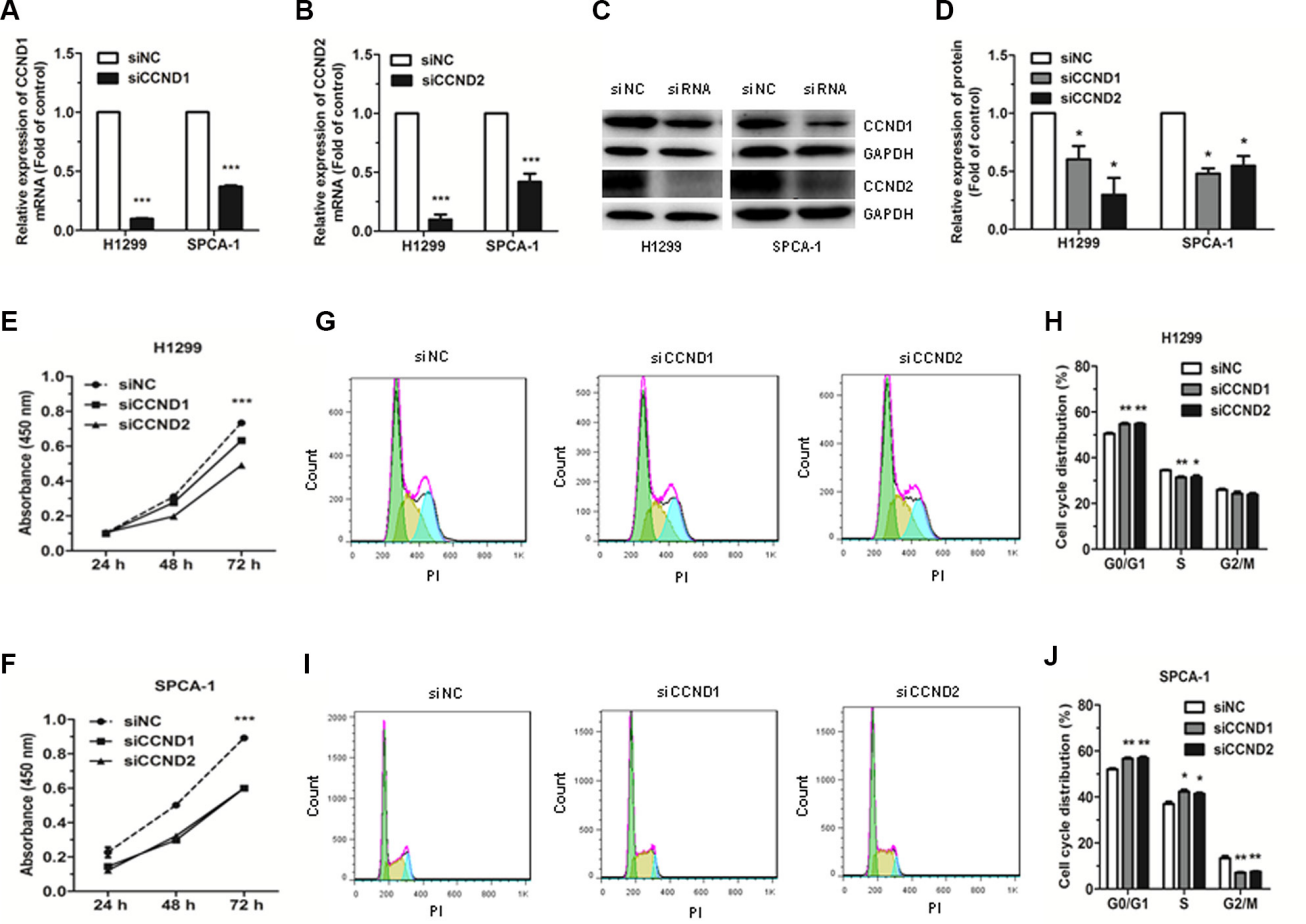
Effects of siRNA targeting of CCND1 and CCND2 on the proliferation and cell cycle of NSCLC cells (**A–B**) The expression of CCND1 and CCND2 mRNA was detected by qRT-PCR in H1299 and SPCA-1 cells transfected with CCND1 and CCND2 siRNAs or siNC. (**C–D)** The protein levels of CCND1 and CCND2 were detected by western blot in H1299 and SPCA-1 cells transfected with CCND1 and CCND2 siRNAs or siNC. (**E–F**) The proliferation of H1299 and SPCA-1 cells transfected with CCND1 and CCND2 siRNAs or siNC, as determined by CCK-8 assay. (**G–J**) Cell cycle distribution was analyzed by flow cytometry in H1299 and SPCA-1 cells transfected with CCND1 and CCND2 siRNAs or siNC. Each experiment was performed in triplicate at least. ^*^P < 0.05, ^**^P < 0.01, ^***^P < 0.001.

To downregulate CCND1 and CCND2 expression and investigate the resulting effects on cell proliferation and cell cycle progression in the NSCLC cell lines, H1299 and SPCA-1, we used siRNAs targeting CCND1 and CCND2. Our data revealed that the mRNA and protein expression levels of CCND1 and CCND2 were significantly downregulated both in H1299 and SPCA- 1 cells transfected with CCND1 and CCND2 siRNAs, as compared with the NC siRNA (siNC) transfected cells (Figure 5A–5D). Results further revealed that the proliferation of cells transfected with CCND1 and CCND2 siRNAs was significantly inhibited compared with the control cells (Figure 5E, 5F). Furthermore, flow cytometric analysis of cell cycle showed that the proportion of cells at G0/G1 phase was significantly increased in cells transfected with CCND1 and CCND2 siRNAs compared with siNC transfected cells (Figure 5G–5J). These results demonstrated that siRNA-mediated downregulation of CCND1 and CCND2 could mimic the effects of miR- 146a-5p upregulation in NSCLC cells.

### MiR-146a-5p inhibits tumor growth *in vivo*

**Figure 6:**
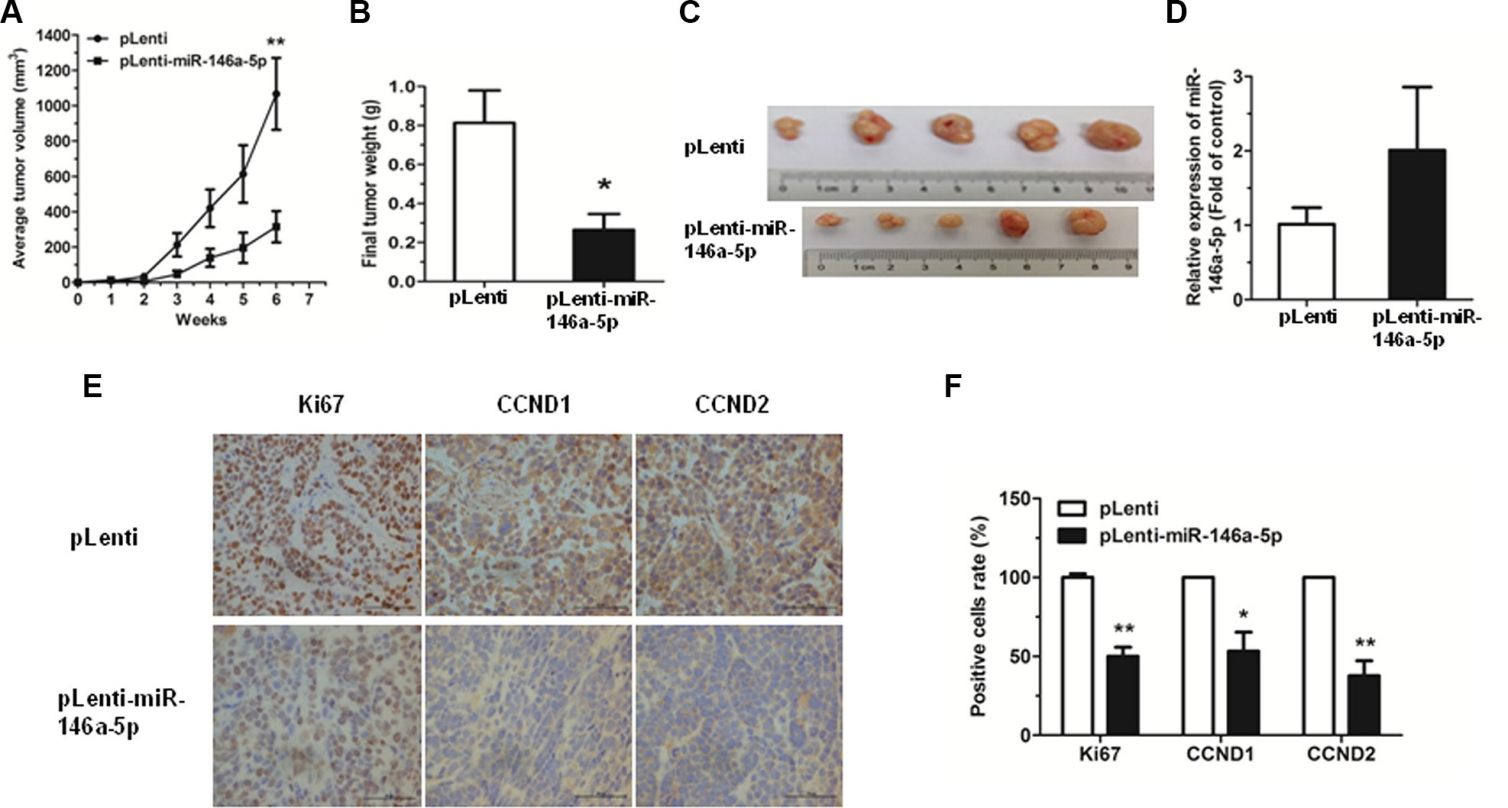
Overexpression of miR-146-5p inhibited tumor growth (**A**) miR-146a-5p-stably-overexpressing SPCA-1 cells (pLenti-miR-146a-5p) and control (pLenti) were injected into nude mice. Each group comprised 5 mice. Tumor sizes were measured every week and growth curves were generated. (**B**) Mice were sacrificed after 6 weeks and tumor weights were measured. (**C)** Tumor images were displayed. (**D)** The expression of miR-146a-5p was detected by qRT-PCR in the mouse tumor tissues induced by negative control and miR-146a-5p-stably-overexpressing SPCA-1 cells. (**E–F)** The expression of Ki67, CCND1 and CCND2 in tumor tissues was measured by immunohistochemistry. ^*^P < 0.05, ^**^P < 0.01.

The antitumor effects of miR-146a-5p were evaluated in a xenograft mouse model. SPCA-1 cells (1 × 10^6^) with stably overexpressing miR-146a-5p (pLenti-miR-146a-5p) or control (pLenti) were injected subcutaneously into nude mice. After 6 weeks, tumor growth was significantly inhibited in the SPCA-1 cells stably overexpressing miR-146a-5p, when compared to the control. The miR-146a-5p-overexpressing cells also exhibited a marked reduction in both tumor size and weight by 6 weeks post-implantation (Figure 6A–6C). Moreover, the expression of miR-146a-5p was significantly upregulated in tumor tissues of pLenti-miR-146a-5p group compared to the controls (Figure 6D). We also measured the expression of Ki67, CCND1 and CCND2 in xenograft tumor tissues using immunohistochemistry. Results showed that Ki67, CCND1 and CCND2 were significantly downregulated in miR-146a-5p-stably-overexpressing tumor tissues (Figure 6E, 6F).

## DISCUSSION

**Figure 7:**
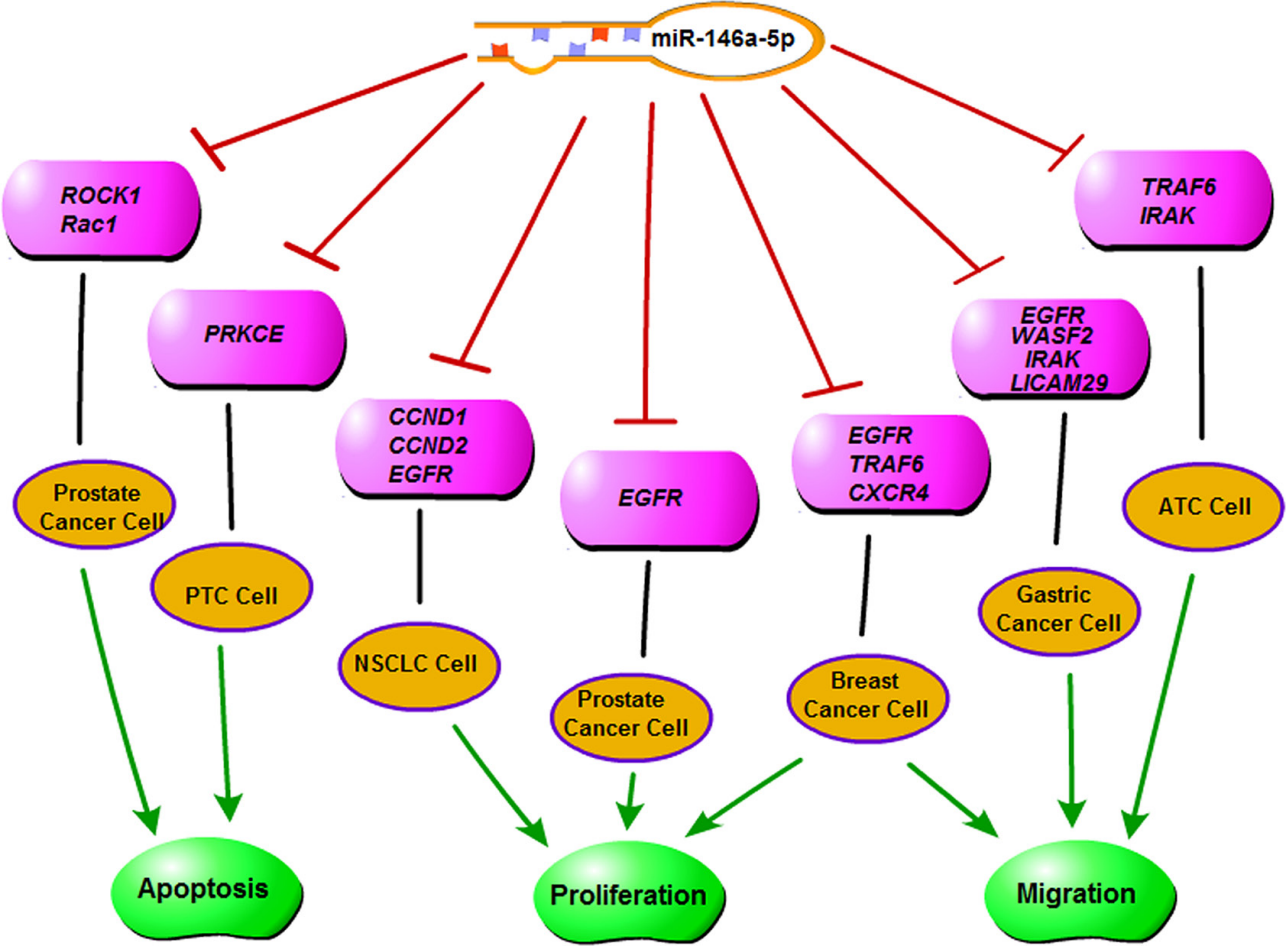
A diagram of the functions of miR-146a-5p

Some miRNAs can target oncogenes, acting as tumor suppressors in some kinds of tumors, yet act as oncogenes in other types of tumors [29, 30]. One of these miRNAs is miR-146a-5p. In papillary thyroid carcinoma and anaplastic thyroid cancer, miR-146a-5p acts as an oncogene by targeting tumor-suppressor genes, thus promoting invasion [13–15] (Figure 7). However, in breast cancer, prostate cancer, pancreatic cancer, gastric cancer, as well as in NSCLC cell lines, miR-146a-5p acted as a tumor suppressor through the targeting of Rac1/ROCK1/EGFR [16–25] (Figure 7). A recent study showed that in the serum of NSCLC patients, miR-146a-5p was significantly lower than in the controls, suggesting its role as a tumor suppressor [31].

In this study, we validated a decrease in miR- 146a- 5p in two NSCLC cell lines as well as in clinical NSCLC samples, and confirmed its role as a tumor suppressor in NSCLC, both *in vitro*, using human cell lines, and *in vivo*, using an engrafted tumor assay. In the NSCLC cell lines, H1299 and SPCA-1, miR-146a- 5p inhibited cell proliferation, colony formation, and caused cell cycle arrest at G0/G1 phase. Additionally, in the miR- 146a-5p-stably-overexpressing SPCA-1 cells xenograft mouse model, tumor growth was also significantly inhibited, compared to the control. To the best of our knowledge, our current study was the first to identify two central cell cycle regulators as bona fide targets of miR-146a-5p, namely, CCND1 and CCND2. Of note, overexpression of CCND1 and CCND2 is one of the most common hallmarks of various cancer types, due to its relatively high amplification frequency (15%–40%) and the upregulation of its mRNA and protein expression in a variety of tumors including NSCLC, breast cancer, melanoma, and pancreatic cancer [32].

As the regulatory subunits of CDK4 or CDK6, CCND1 and CCND2 are required for cell cycle G1/S transition, and thus have been implicated as proto-oncogenes and attractive targets for cancer therapy. Previous studies have shown that CCND1 is the direct target of miR-186 [33] and miR-545 [34], while both CCND1 and CCND2 are directly targeted by miR-15a and miR-16 [35].

In conclusion, our results demonstrated that miR- 146a-5p acted as a tumor suppressor through directly targeting CCND1 and CCND2 in the NSCLC cell lines, H1299 and SPCA-1. This finding identifies miR-146a- 5p as a promising candidate for molecular therapy in the treatment of NSCLC.

## MATERIALS AND METHODS

### Tissue samples and ethics statement

Human lung cancer samples were obtained from the Department of Oncology, Shanghai Chest Hospital (Shanghai, China) with the informed consents of patients and approval by the Ethics Committee of Shanghai Chest Hospital. The clinicopathological factors of patients were shown as the published paper [36].

### Cell culture and cell transfection

SPCA-1, 95-D, HEK293T, and BEAS-2B cells were obtained from the Cell Bank, China Academy of Sciences (Shanghai, China). H1299 and H23 cells were purchased from the American Type Culture Collection (ATCC, Manassas, VA, USA). The BEAS-2B cell line was isolated from normal human bronchial epithelium. H1299 is p53^−/−^ NSCLC cell line, SPCA-1 is Asian NSCLC cell line, H23 is KRAS mutation NSCLC cell line and 95-D is large cell lung cancer (LCLC) with high migration.

The NSCLC cell lines, H1299, H23, 95-D, and SPCA-1 were cultured in either RPMI-1640 medium or Dulbecco's modified Eagle's medium (DMEM, Gibco, Gaithersburg, MD, USA). BEAS-2B cells were cultured in LHC-9 medium and HEK293T cells were cultivated in DMEM (Gibco). All media were supplemented with 10% fetal bovine serum (FBS, HyClone Laboratories, Logan, UT, USA), and antibiotic cocktail, 100 U/ml penicillin and 100 μg/ml streptomycin (Gibco). Cell culture was carried out at 37°C in a 5% CO_2_ humidified environment.

Cells were transiently transfected with 100 nM of chemically synthesized siCCND1-2, siCCND2-1 (Table 1), or negative control siRNA (siNC, Ribobio, Guangzhou, China) using Lipofectamine 2000 (Invitrogen), according to the manufacturer's instructions. After 24 to 48 h post-transfection, cells were used for subsequent experiments including assays for cell proliferation, cell cycle analysis, qRT-PCR, and western blotting.

**Table 1:** Sequences of siRNAs for CCND1 and CCND2

Name	Target mRNA	Sequence (5′–3′)
siCCND1 (-1)	CCND1	UGGAAUAGCUUCUGGAAUU
siCCND1 (-2)	CCND1	CCGCACGAUUUCAUUGAAC
siCCND1 (-3)	CCND1	GCAUGUUCGUGGCCUCUAA
siCCND2 (-1)	CCND2	CGGAGAAGCUGUGCAUUUA
siCCND2 (-2)	CCND2	GAUCCAAGUCGGAGGAUGA
siCCND2 (-3)	CCND2	UGAGCUCGCUCACUUGUGA

### qRT-PCR analysis

Total RNA was extracted from the cells using TRIzol Reagent (Sangon Biotech, Shanghai, China), according to the manufacturer's instruction, and then stored at -80°C for further study. Reverse transcription was performed using the PrimeScript^™^ 1st Strand cDNA Synthesis Kit (M-MLV RTase cDNA Synthesis Kit, TaKaRa, Dalian, China), and a cDNA library of miRNAs was synthesized using the PrimeScript**®** miRNA First-Strand cDNA Synthesis SuperMixQuantiMir cDNA Kit (Transgen Biotec, Beijing, China). The level of mRNA or miRNA was quantified by qRT-PCR using a SYBR Green PCR master mix (TaKaRa). The endogenous controls for mRNA and miRNA were 18S RNA and U6 snRNA, respectively. Results were expressed using relative quantification (2^-ΔΔCt^) method. Primer sequences are shown in Table 2.

**Table 2:** Primer sequences used for amplification

Name	Usage	Sequence (5′–3′)
18S RNA	qPCR forward	AGGAATTCCCAGTAAGTGCG
	qPCR reverse	GCCTCACTAAACCATCCAA
U6 snRNA	qPCR forward	CTCGCTTCGGCAGCACA
	qPCR reverse	AACGCTTCACGAATTTGCGT
pri-miR-146a	PCR forward	CGGATCCTTGGTCTCCTCCAGATGTTTAT
	PCR reverse	CCTCGAGTCATTAAAGTGATTTCTCCCAAG
CCND1	qPCR forward	GGCGGAG GAGAACAAACAGA
	qPCR reverse	ATGGAGGGCGGATTGGAAA
CCND2	qPCR forward	TCCAAACTCAAAGAGACCAGC
	qPCR reverse	TTCCACTTCAACTTCCCCAG
CCND1-3′-UTR	PCR forward PCR reverse	GCTCTAGA GTTTGGCGTTTCCCAGAGT GGATATC ATGGCTAAGTGAAGCATGAGG
CCND2- 3′-UTR	PCR forward PCR reverse	GCTCTAGA CTGGGTTACTCTTCGCTTCTG GGATATC GAAACTGTACGGTTGTGAGGTATT
CCND1- 3′-mUTR	PCR forward PCR reverse	TTAATCTCCTCTTAGAACATTGTATTACAGATGC GAGGAGATTAAAACTAGTACATGGCAGTATATG
CCND2- 3′-mUTR	PCR forward PCR reverse	CATATTCCGGGTGTTCCTACCAGGACTCAAGA CCCGGAATATGTACCAAAGAACGCCAGATACC

### Cell proliferation assay

Cell proliferation was determined with CCK-8 assay (Dojindo, Japan). Briefly, cells were plated in a 96-well plate at a density of 2×10^3^ cells per well and incubated at 37°C in a 5% CO_2_ humidified environment. CCK-8 was added and cells were returned to incubation conditions for 1–4 h. Light absorbance at 450 nm was measured daily with a microplate reader. Experiments with triplicates were performed independently at least thrice.

### Colony formation assay

Cells were plated at 300 cells per 35-mm tissue culture dish and incubated at 37°C in a 5% CO_2_ humidified environment for 2 weeks. Colonies were then fixed with methanol, stained with crystal violet (0.5% w/v), and counted. Experiments with duplicates were performed independently at least thrice.

### Cell cycle analysis

Cell cycle analysis was performed as previously described [37]. In brief, cells (1×10^5^) were collected and fixed in 75% ethanol at -20°C overnight. The cells were then harvested, treated with RNase A (100 ng/ mL) for 30 min, and stained with propidium iodide (PI) (50 ng/ mL) for 15 min. After staining, samples were analyzed for cell cycle distribution with a MoFlo XDP flow cytometer (Beckman Coulter, Inc., Brea, CA, USA). Data were analyzed using Flow Jo software (Treestar Inc., USA). Experiments with duplicates were performed independently at least thrice.

### Dual luciferase reporter assay

Target genes for miR-146a-5p were selected based on Starbase (http://starbase.syst.edt.cn/). The 3′ -UTR of the target genes (CCND1 and CCND2) were sub-cloned downstream of the firefly luciferase reporter gene in the pGL3 miReport vector (Promega, Madison, WI, USA). The recombinant vectors for CCND1 and CCND2 were named pGL3-CCND1-3′-UTR and pGL3-CCND2-3′-UTR, respectively. Mutant 3′-UTRs for CCND1 and CCND2, each containing a mutated binding site for miR-146a-5p, were cloned into pGL3 and named as pGL3-CCND1-3′-mUTR and pGL3-CCND2-3′-mUTR, respectively. Recombinant expression vectors were confirmed by sequencing (Sangon Biotech). The primer sequences are listed in Table 2.

Dual luciferase reporter assays were performed as previously described [36, 37]. In brief, HEK293T cells were cultured in 24-well plates and transiently co-transfected with 400 ng of luciferase vector pGL3-3′-UTR or pGL3-3′-mUTR, and miR-146a-5p mimic or miRNA NC, at a final concentration of 100 nM with 20 ng plasmid expressing the renilla luciferase gene (pRL, Promega) as the control for transfection efficiency. Cells were lysed 48 h after transfection and luciferase activity was assayed with an Orion II Microplate Illuminometer (Titertek-Berthold, South San Francisco, USA). Relative activities were expressed as the fold-change in luciferase activity after normalization to renilla luciferase activity.

### Western blot analysis

Western blot analysis was performed as previously described [36, 37]. In brief, total protein was extracted from the cells using RIPA lysis buffer (CWBIO, Beijing, China) and quantified with a Protein BCA Assay Kit (Bio-Rad, Hercules, California, USA). The protein was then separated by sodium dodecyl sulfate-polyacrylamide gel electrophoresis (SDS-PAGE) and transferred to a polyvinylidene difluoride (PVDF) membrane (Millipore Corporation, Billerica, MA, USA). The PVDF membrane was then blocked in 5% powdered milk at room temperature for 1 h followed by incubation with rabbit anti-CCND1, anti-CCND2, and anti-GAPDH antibodies (1:1000, Cell Signaling Technology, Danvers, MA, USA) overnight at 4°C. After washing and incubation with a goat-anti-rabbit secondary antibody conjugated to horseradish peroxidase (HRP) (1:1000, Cell Signaling Technology), protein bands were detected by a chemiluminescent HRP substrate (Millipore) and imaged with an E-Gel Imager.

### Lentivirus construction and infection

H1299 genomic DNA was used to PCR amplify the pri-miR-146a-5p with its native flanking sequence (99 bp), which was then double digested with BamH I and Xhol I and cloned into the lentiviral expression vector, pLenti (Invitrogen, Carlsbad, CA, USA), to form pLenti-miR-146a-5p. The recombinant expression vector was confirmed by sequencing (Sangon Biotech). To generate lentiviral particles, pLenti-miR-146a-5p or pLenti vector was co-transfected into HEK293T cells using a packaging plasmid system (psPAX2 and pMD2G) and viral particles were collected 24 h and 48 h later. H1299 and SPCA-1 cells were then infected with the viral particles for 48 h. The cells transfected with pLenti-miR-146a-5p or pLenti were sorted for green fluorescence via flow cytometry (Beckman Coulter) to establish stable overexpression of miR-146a-5p (pLenti-miR-146a-5p) or negative control (NC, pLenti) in the H1299 and SPCA-1 cell lines. Cells were expanded and harvested for further experiments.

### Tumor xenograft assay

Female nude mice (six-week-old) were purchased from the SLRC Laboratory Animal Center (Shanghai, China) and maintained under specific-pathogen-free conditions. The subcutaneous tumor xenograft model was established as previously described [38]. Briefly, animals were randomly assigned into 2 groups, and each mouse was injected subcutaneously in the right flank with 1 × 10^6^ SPCA-1 cells stably overexpressing miR-146a-5p (pLenti-miR-146a-5p) or control (pLenti). The tumor diameter was measured weekly with calipers and the tumor volume was calculated using the following formula: volume = length × width^2^/2. Upon conclusion of the experiment, the mice were sacrificed and xenograft tumors were excised and weighed. All experimental protocols were approved by the institutional Animal Care and Use Committee of Shanghai University (Shanghai, China).

### Immunohistochemical determination of tumor growth and miR-146a-5p target genes

The tumor growth was determined by immunohistochemical staining of Ki67 to quantitate growth index, and miR-146a-5p target genes, CCND1 and CCND2, were also measured by immunohistochemistry. Tumor biopsies were fixed by formalin, embedded by paraffin, and finally cut in sections of about 4 μm. Samples were deparaffinized and dehydrated with xylene and graded alcohols, and subsequently rehydrated with demineralized water. Immunohistochemistry was performed using microwave pre-treatment of slides for antigen retrieval. The primary antibodies against Ki67, CCND1 and CCND2 (1:500, Cell Signaling Technology) were applied, together with goat anti-rabbit horseradish peroxidase (HRP)-conjugated antibodies, and the proteins *in situ* were visualized by 3, 3′-diaminobenzidine reaction solution.

### Statistical analysis

Results were expressed as group means ± SEM and analyzed with student′s *t*-test for 2-group comparisons. Differences were considered statistically significant when *p* < 0.05.

## SUPPLEMENTARY MATERIALS FIGURES


