# Supplementary material for: MiR-146a-5p inhibits cell proliferation and cell cycle progression in NSCLC cell lines by targeting CCND1 and CCND2
Source: Oncotarget. 2016 Aug 3;7(37):59287–98. doi: 10.18632/oncotarget.11040 (PMC5312312; doi:10.18632/oncotarget.11040)
Supplement: Supplementary file 1 [file oncotarget-07-59287-s001.pdf]

MiR-146a-5p inhibits cell proliferation and cell cycle progression in NSCLC cell lines by targeting CCND1 and CCND2

Supplementary Materials

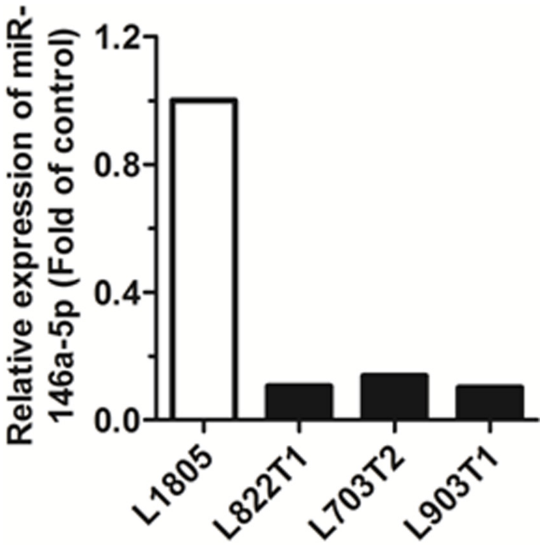

Supplementary Figure S1: The expression of miR-146a-5p in lung tissues of the normal mouse and NSCLC mouse models.

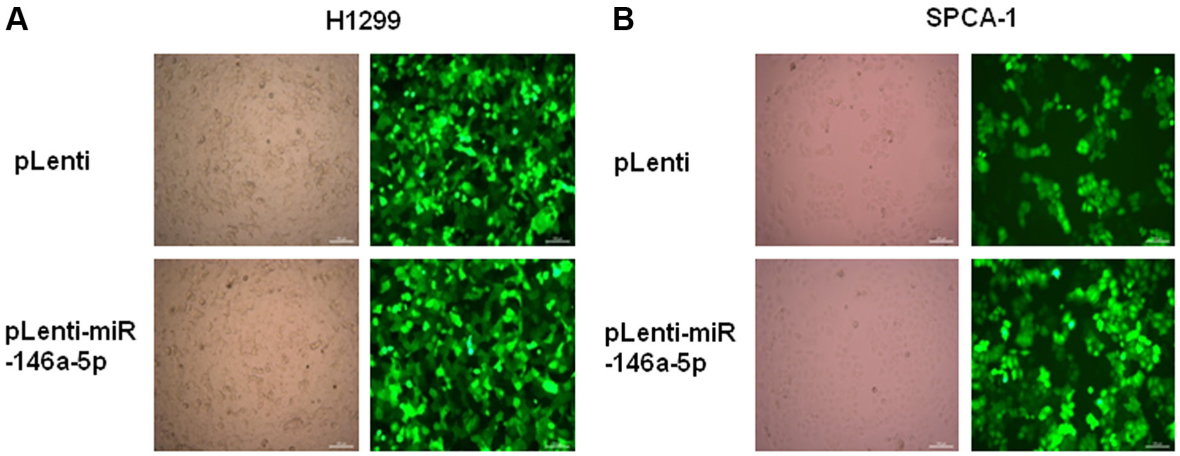

Supplementary Figure S2: Green fluorescence of nearly 99% in both the control (pLenti) and miR-146a-5p-stably-overexpressing NCSCL cell lines was observed under fluorescence microscope. (**A**) H1299 cell line. (**B**) SPCA-1 cell line.
